# Mechanisms of dihydrotestosterone action on resveratrol-induced anti-proliferation in breast cancer cells with different ERα status

**DOI:** 10.18632/oncotarget.5482

**Published:** 2015-10-06

**Authors:** Yu-Tang Chin, Sheng-Huei Yang, Tung-Cheng Chang, Chun A. Changou, Hsuan-Yu Lai, Earl Fu, Wei-Chun HuangFu, Paul J. Davis, Hung-Yun Lin, Leroy F. Liu

**Affiliations:** ^1^ Taipei Cancer Center, Taipei Medical University, Taipei, Taiwan; ^2^ PhD program for Cancer Biology and Drug Discovery, College of Medical Science and Technology, Taipei Medical University, Taipei, Taiwan; ^3^ Division of Colorectal Surgery Department of Surgery, Taipei Medical University-Shuang-Ho Hospital, New Taipei City, Taiwan; ^4^ Integrated Laboratory, Center of Translational Medicine, Taipei Medical University, Taipei, Taiwan; ^5^ Core Facility, Taipei Medical University, Taipei, Taiwan; ^6^ Department of Periodontology, School of Dentistry, National Defense Medical Center and Tri-Service General Hospital, Taipei, Taiwan; ^7^ Pharmaceutical Research Institute, Albany College of Pharmacy and Health Sciences, Rensselaer, New York, USA; ^8^ Albany Medical College, Albany, New York, USA

**Keywords:** resveratrol, DHT, estrogen receptor-α, integrin αvβ3, breast cancer

## Abstract

Dihydrotestosterone (DHT) has been shown to promote breast cancer growth via different mechanisms. In addition to binding to ERα, the DHT membrane receptor exists on integrin αvβ3. Resveratrol induces p53-dependent apoptosis via plasma membrane integrin αvβ3. Resveratrol and DHT signals are both transduced by activated ERK1/2; however, DHT promotes cell proliferation in cancer cells, whereas resveratrol is pro-apoptotic. In this study, we examined the mechanism by which DHT inhibits resveratrol-induced apoptosis in human ERα positive (MCF-7) and negative (MDA-MB-231) breast cancer cells. DHT inhibited resveratrol-stimulated phosphorylation of Ser-15 of p53 in a concentration-dependent manner. These effects of DHT on resveratrol action were blocked by an ERα antagonist, ICI 182,780, in MCF-7 breast cancer cells. DHT inhibited resveratrol-induced nuclear complex of p53-COX-2 formation which is required p53-dependent apoptosis. ChIP studies of COX-2/p53 binding to DNA and expression of p53-responsive genes indicated that DHT inhibited resveratrol-induced p53-directed transcriptional activity. In addition, DHT did inhibit resveratrol-induced COX-2/p53-dependent gene expression. These results suggest that DHT inhibits p53-dependent apoptosis in breast cancer cells by interfering with nuclear COX-2 accumulation which is essential for stimulation of apoptotic pathways. Thus, the surface receptor sites for resveratrol and DHT are discrete and activate ERK1/2-dependent downstream effects on apoptosis that are distinctive. These studies provide new insights into the antagonizing effects of resveratrol versus DHT, an important step toward better understanding and eventually treating breast cancer. It also indicates the complex pathways by which apoptosis is induced by resveratrol in DHT-depleted and -repleted environments.

## INTRODUCTION

Androgens have important physiological effects on women [1]. They are the precursor hormones for estrogen biosynthesis in the ovaries and extragonadal tissues [2] and also function directly via androgen receptors (ARs) throughout the body [3]. AR is expressed in normal breast cells, and up to 85% of breast tumors are AR positive [4]. Yeh *et al* indicate that AR plays an important role in breast cancer proliferation [5]. However, stimulation with dehydroepiandrosterone sulfate (DHEAS) induces breast cancer cell proliferation through the ER [6, 7], but inhibits proliferation through AR. Studies show that plasma testosterone concentrations appear to be associated with increased breast cancer risk among postmenopausal hormone users [8]. Other epidemiological studies also suggest that plasma levels of testosterone are significantly associated with breast cancer risk in pre- and postmenopausal women [9]. Furthermore, high baseline levels of serum testosterone have emerged as a strong prognostic factor for contralateral breast cancer, distant metastasis and local relapse [10], although it is debatable that testosterone effects on breast cancer progression could also result from conversion to 17β-estradiol (E_2_) via aromatization in peripheral tissues [5]. Thymidine uptake studies also indicate that the non-aromatizable androgen metabolite, dihydrotestosterone, DHT-induced [^3^H]-thymidine incorporation can be inhibited by ICI 182,780, a pure anti-estrogen that serves as an antagonist of the ER in ER-α-positive MCF-7 breast cancer cells [11, 12] in which other ERs such as ER-β, GPR30 and ER-α spliced variants such as ER-α36, and ER-α46 also exist [13–16]. On the other hand, the action of DHT is blocked by RGD peptide which blocks the binding site on integrin αvβ3 in ER-α-negative MDA-MB-231 breast cancer cells which contain ER-α36 and GPR30 [13]. Although ER-α36 [13, 14] GPR30 [15] and ER-β [16] have been shown to play roles in cell proliferation, the mechanisms involved are not fully understood.

Studies also indicate that the proliferative signal induced by DHT is transduced by discrete mechanisms in ER-α-positive and ER-α-negative breast cancer cells [11]. Interestingly, the role of integrin αvβ3 on ER-α-positive breast cancer MCF-7 cells is controversy, although it is reported that there is no integrin αvβ3 existing on MCF-7 cells [17, 18] and others suggest integrin αvβ3 exists on MCF-7 cells [19].

Phosphoinositide 3-OH kinases (PI3Ks) are a group of major intracellular signaling molecules [20] whose activation has been shown to be involved in proliferation and development of tumors [21]. Estrogen activates PI3K/Akt and ERK1/2 signalings through ER-α-dependent mechanism which is involved in cell proliferation in breast cancer cells [22–24]. Inhibition of PI3K also inhibits cancer growth [22, 25–27].

Resveratrol (3, 5, 4′-trihydroxy-trans-stilbene) is a naturally occurring trihydroxyl-diphenylethylene compound which has beneficial effects in the treatment of cancer and cardiovascular disease [28–31]. It inhibits carcinogen-induced pre-neoplastic lesions and mammary tumors in rodent models [32]. Resveratrol is able to bind to and to activate ER but with far lower affinity than E_2_ does [33]. As other selective ER modulators such as tamoxifen, resveratrol has been considered to have potential as an anti-breast cancer adjunct [32]. Although mechanisms involved in the resveratrol-induced anti-proliferation of cancers are not fully understood, recently, we have shown that resveratrol induces anti-proliferation via integrin αvβ3 [34] binding site to activate ERK1/2, to induce nuclear accumulated cyclooxygenase-2 (COX-2) and p53-dependent mechanism in breast cancer, glioma, head and neck squamous cell cancer and ovarian cancer cells [29, 30, 35, 36]. The nuclear accumulated COX-2 forms complex with phosphorylated p53 and ERK1/2 as a co-activator for p53-responsive genes [35, 36].

In the present study, we investigate the hypothesis that both resveratrol and DHT induced ERK1/2 activation and led to the disparate effects via different receptors in ER-α-positive and negative breast cancer cells. In ER positive breast cancer MCF-7 cells, DHT bound to ER and it bound to integrin αvβ3 in ER-negative MDA-MB cells. However, resveratrol bound to integrin αvβ3 in both types of cancer cells. While DHT stimulates breast cancer cell proliferation, the nuclear accumulation of COX-2 and p53-dependent action of resveratrol induces anti-proliferation. Resveratrol-associated apoptosis requires inducible accumulation of nuclear COX-2 upstream of p53. The inhibition of apoptosis by DHT in resveratrol-treated cells is initiated at the activated ERK1/2 after DHT binds to integrin receptor on the cell surface for the hormone; this leads to suppress the nuclear interaction of COX-2 protein and activated ERK1/2, which is essential to the pro-apoptotic action of resveratrol. This nuclear complex is formed in breast cancer cells exposed to resveratrol, alone, but does not occur in DHT-treated cells or in cells incubated with both resveratrol and DHT.

## RESULTS

### DHT interferes with resveratrol-induced anti-proliferation in breast cancer cells

**Figure 1:**
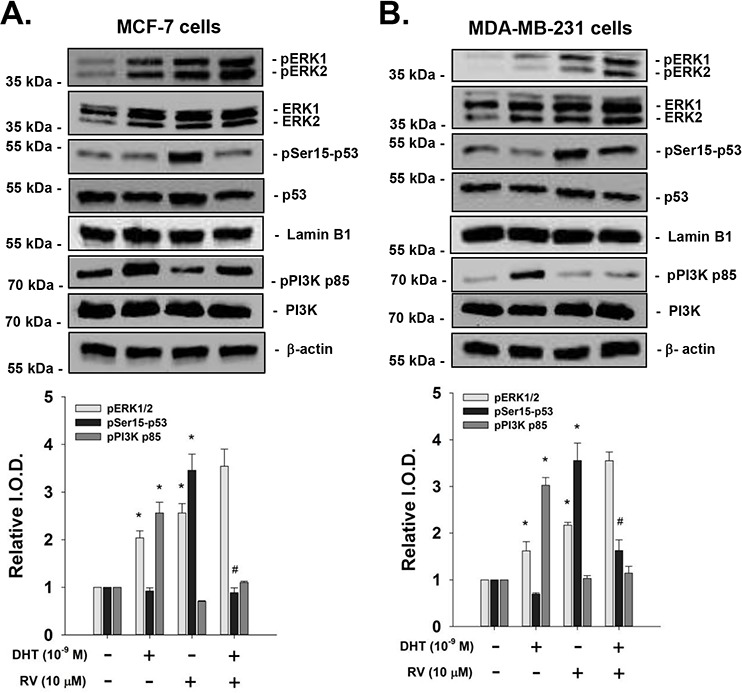
Effect of DHT on resveratrol-induced p53 phosphorylation in breast cancer cells Human breast cancer MCF-7 cells **A.** and MDA-MB-231 cells **B.** were treated with 10 μM resveratrol in the presence or absence of 10^−9^ M DHT for 4 h. Both DHT and resveratrol activated ERK1/2 and DHT activated PI3K. DHT inhibited RV-induced p53 phosphorylation and resveratrol reduced DHT-induced PI3K activation. Lamin-B1 was used as a nuclear internal loading control. β-actin was used as a cytosolic internal loading control. Number of independent experiments (N) = 3. *p < 0.05, compared to control; #*p* < 0.05 compared between RV-treated samples in the presence or absence of DHT. (RV: resveratrol)

**Figure 2:**
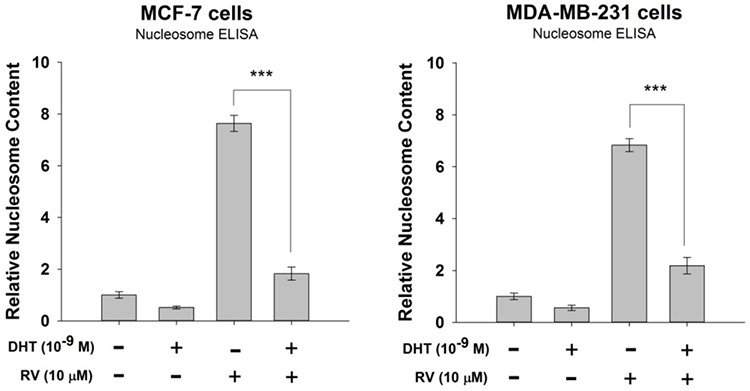
Effect of DHT on resveratrol-induced anti-proliferation in breast cancer cells **A.** Human breast cancer MCF-7 cells were treated with 10 μM resveratrol in the presence or absence of 10^−9^ M DHT for 48 h. Apoptosis was measured by Nucleosome ELISA. *N* = 3. ***p < 0.001, compared between RV-treated samples in the presence or absence of DHT **B.** Human breast cancer MBA-MD cells were treated with 10 μM resveratrol in the presence or absence of 10^−9^ M DHT for 48 h. Apoptosis was measured by Nucleosome ELISA. *N* = 4. ***p < 0.001, compared between RV-treated samples in the presence or absence of DHT.

When human breast cancer MCF-7 cells were treated with 10 μM resveratrol, there was activation of ERK1/2 and Ser-15 phosphorylation of p53. DHT (10^−9^M) induced activation of ERK1/2, in addition, DHT also activated PI3K but not phosphorylation of p53. In the presence of DHT, resveratrol-induced Ser-15 p53 phosphorylation was inhibited by DHT in MCF-7 cells (Fig. 1A). However, ERK1/2 activated by resveratrol and DHT was additive. Resveratrol also induced ERK1/2 activation and Ser-15 p53 phosphorylation in ER-negative breast cancer MDA-MB-231 cells (Fig. 1B). On the other hand, DHT induced ERK1/2 and PI3K but not p53 activation (Fig. 1B). The activation of p53 by resveratrol was inhibited by co-treatment of DHT and the activation of PI3K by DHT was blocked by resveratrol co-treatment but the ERK1/2 activation was not inhibited by the co-treatment (Fig. 1B). Furthermore, resveratrol-induced apoptosis in breast cancer MCF-7 cells was also inhibited by DHT treatment (Fig. 2A). Similar results were observed in ER-α-negative breast cancer MBA-MB cells (Fig. 2B).

**Figure 3:**
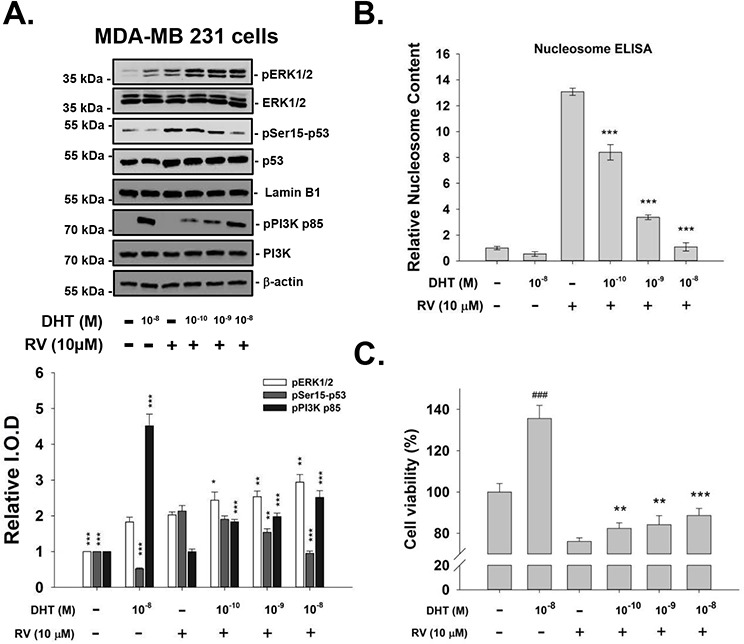
DHT inhibits resveratrol-induced p53 phosphorylation and subsequent apoptosis, in a concentration-dependent manner **A.** Human breast cancer MDA-MB-231 cells were treated with DHT (10^−10^–10^−8^ M) with or without 10 μM resveratrol for 4 h. Immunoblots of nuclear fractions were from this representative experiment. Bar graph shows that RV-induced ERK1/2 activation (pERK1/2, top panel, lane 3) was enhanced by 10^−10^–10^−8^ M DHT. However, RV-induced Ser-15 phosphorylation of p53 was inhibited by DHT in a dose-dependent manner, with the most marked inhibition occurring at 10^−8^ M DHT. *N* = 4. *p < 0.05; **p < 0.005, compared to control. **B.** The extent of apoptosis measured by Nucleosome ELISA in MDA-MB-231 cells were treated with DHT (10^−10^–10^−8^ M) with or without 10 μM resveratrol for 24 h. Resveratrol alone caused apoptosis, but DHT did not. DHT inhibited nucleosome formation induced by resveratrol in a dose-dependent manner. *N* = 5. *p < 0.05; **p < 0.005, compared to resveratrol treatment. **C.** The cell viability of MCF-7 was measured by using MTT assay. Cells were treated with DHT (10^−10^–10^−8^ M) with or without 10 μM resveratrol for 4 days with reflashed medium and treatment daily. Resveratrol alone caused anti-proliferation to ~80%, but DHT enhanced cell proliferation to ~140%. DHT reversed anti-proliferation induced by resveratrol in a dose-dependent manner. *N* = 6. ### *p* < 0.05, compared to control; *p < 0.05; **p < 0.005, ***p < 0.001, compared to resveratrol treatment.

We also studied the concentration effect of DHT on resveratrol-induced signals. MDA-MB-231 cells were treated with resveratrol in the presence or absence of DHT (10^−10^–10^−8^ M) for 4 h. Resveratrol-induced Ser-15 phosphorylation of p53 was inhibited by DHT in a concentration-dependent manner (Fig. 3A). The effect of 10^−10^–10^−8^ M DHT on resveratrol-induced anti-proliferation in MDA-MB-231 cells, indicated by nucleosome ELISA, is shown in Fig. 3B. The similar findings that DHT reduced resveratrol-induced anti-proliferation dose-dependently were also observed in MCF-7 cells by using MTT assay (Fig. 3C). Dose-responsive inhibition by DHT of anti-proliferation in these resveratrol-treated cells correlates directly with the reduction in phosphorylation of Ser-15 p53 seen above (Fig. 3A).

### DHT interferes with resveratrol-induced anti-proliferation via different binding sites

**Figure 4:**
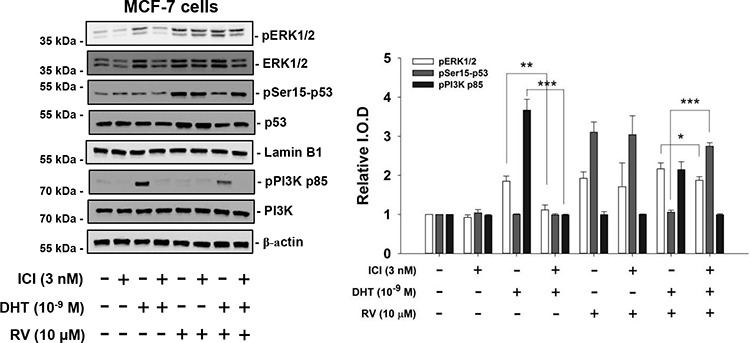
ICI 182,780 (ICI) inhibits the effects of DHT on resveratrol-induced activation of ERK1/2 and p53 in MCF-7 cells MCF-7 cells were pretreated with 3 nM ICI or diluent for 30 min, after which they were treated with 10^−9^ M DHT and/or 10 μM resveratrol for 4 h along with continued 3 nM ICI or diluent as in the pretreatment period. Results shown are representative of three experiments. Immunoblots of nuclear fractions show that ICI minimally inhibited DHT-induced ERK1/2 activation (comparing lanes 3 and 4), but did not inhibit RV-induced ERK1/2 activation (lanes 5 and 6). The additive effect on ERK1/2 activation of DHT and resveratrol was reduced by ICI, and the inhibitory effect of DHT on RV-induced serine phosphorylation of p53 was partially reversed by ICI treatment. *N* = 3. *p < 0.05; **p < 0.005; ***p < 0.001, the comparisons are as indicated.

Previously we have shown that DHT binds to different binding sites in breast cancer cells to activate signal transduction pathway, which activates ERK1/2 and cell proliferation [11]. It binds to ERα in ERα positive breast cancer MCF-7 cells and to integrin αvβ3 in ER negative MDA-MB-231 cells. In order to investigate the mechanisms involved in DHT-induced inhibitory effects on resveratrol-induced anti-proliferation in human breast cancer cells, studies have been designed to treat ERα-positive with resveratrol and DHT in the presence or absence of ERα antagonist, ICI182,780 [37]. Both DHT and resveratrol induced ERK1/2 activation (Fig. 4). DHT inhibited resveratrol-induced phosphorylation of Ser-15 of p53. In the presence of ICI, the effect of resveratrol was not affected but the DHT-induced actions were inhibited including the inhibitory effect on resveratrol (Fig. 4). ICI did not inhibit resveratrol-induced Ser-15 phosphorylation of p53. On the other hand, DHT blocked Ser-15 phosphorylation of p53 induced by resveratrol, but in the presence of DHT and ICI, the inhibitory effect of the hormone on phosphorylation of p53 was not seen. The action on ERK1/2 activation by DHT alone was inhibited by ICI. The inhibitory effect of DHT on resveratrol-induced Ser-15 phosphorylation of p53 appears to be ER-mediated, in that this hormone effect was reversed by the ER inhibitor.

**Figure 5:**
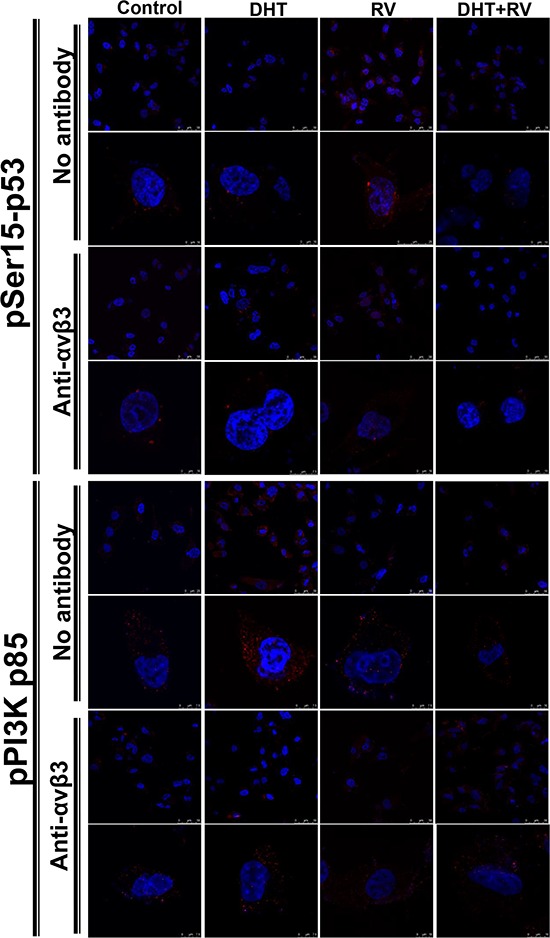
Blockage of integrin αvβ3 activity by antibody inhibits DHT-induced PI3K activation in resveratrol-treated MDA-MB-231 cells MDA-MB-231 cells were pre-treated with anti-integrin αvβ3 antibody for 24 h prior to treatment of 10^−9^ M DHT or 10 μM resveratrol for another 24 h. The cells were stained for anti-pSer15-p53 antibody or anti-phosphoPI3K (pPI3K p85) antibody, and Hoechst stain for nucleus. These merged images were presented by using confocal microscopy. Treatment of resveratrol induced nuclear translocation of pSer15-p53, but this action was blocked when integrin αvβ3 was neutralized. The expressions of pPI3K were induced by DHT in cytosol, but were attenuated by integrin αvβ3 neutralization. The scale bar was presented on the right bottom of each image.

Studies were also conducted in ERα negative MDA-MB-231 cells. DHT, RV or the combination was added to cells treated with 0.5 μg/ml of anti-integrin αvβ3 antibody for 24 h previously. Resveratrol induced p53 phosphorylation and nuclear translocation which was inhibited by co-treatment with DHT. The DHT induced PI3K activation which was inhibited by resveratrol. In the pretreatment of anti-integrin antibody, the resveratrol-induced p53 phosphorylation and the DHT-induced PI3K activation were blocked by the antibody (Fig. 5). These results suggest that resveratrol and DHT share integrin αvβ3 as their binding sites in ERα negative MDA-MB-231 cells.

### DHT inhibits resveratrol-induced nuclear accumulation of COX-2, binding of p53 to DNA and p53-dependent gene expression

**Figure 6:**
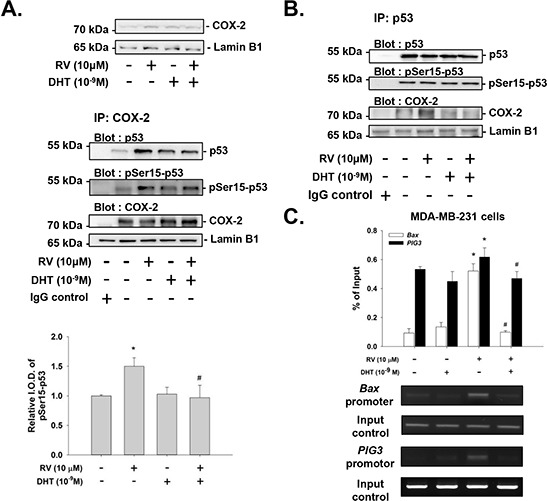
Effects of DHT on resveratrol-induced complexing of COX-2 with pSer-15-p53 and DNA binding in MDA-MB-231 cells Cells were treated with resveratrol with or without DHT (10^−9^M) for 24 h. **A.** Nuclear proteins were isolated and Western blot analysis was conducted to examine resveratrol-induced nuclear COX-2 accumulation (upper pannel). Nuclear extracts were also immunoprecipitated with anti-COX-2 antibody. Western blot analyses were performed with antibodies against p53, pSer15-p53 and COX-2. Resveratrol induced increased nuclear COX-2 which was inhibited by co-incubation with DHT. The accumulation of complex with COX-2 and p53 was not increased by DHT alone, but was stimulated by resveratrol, along with complexing with pSer15-p53. These latter effects of resveratrol were inhibited by DHT. Lamin-B1 was served as loading control for immunoprecipitation of nuclear proteins. The lower panel was shown for quantification of p53 which bound with COX-2. **B.** The similar results were observed in immunoprecipitation with p53 antibody. **C.** Cells were fixed and harvested for ChIP and immunoprecipitaed with COX-2 antibody as described in Materials and Methods. The specific primers for p53 responsive DNA promoter sequence, *Bax* and *PIG3*, were analized by QPCR. Results of PCR were also presented. The resveratrol induced p53-responsive DNA promoter binding was attenuated by DHT pretreatment. *N* = 3. *p < 0.05, compared to control; # *p* < 0.05, compared between resveratrol-treated samples in the presence and absence of DHT.

Resveratrol induces nuclear accumulation of COX-2 which facilitates resveratrol-induced p53-dependent apoptosis [29, 30, 35, 36]. Treatment of MDA-MB-231 cells with resveratrol in the presence of DHT resulted in not only reducing the nuclear tanslocation of COX-2, but also decreasing the nuclear complex of COX-2 and p53 (Fig. 6A). Similar complexing between COX-2 and p53 was observed when nuclear proteins were immunoprecipitated with anti-p53 antibody (Fig. 6B).

p53 and COX-2 have been shown to bind to p53-responsive gene promoter sequences [35, 36, 38]. Estrogen inhibits resveratrol-induced apoptosis by inhibiting p53-dependent gene expression in human breast cancer cells [38]. The androgen may show the same inhibition of resveratrol-induced anti-proliferation by blocking promoter-binding activity. To determine whether p53-responsive gene promoter activity is affected by androgen, chromatin immunoprecipitation (ChIP) experiments were performed in MDA-MB-231 cells. Results shown in Fig. 6C indicate that resveratrol induced nuclear COX-2 to bind to the p53-responsive gene promoter, *Bax* and *PIG3*. Further, DHT inhibited interaction of COX-2 with p53 (Fig. 6A and 6B) and the promoter DNA sequence (Fig. 6C). Therefore, DHT inhibits not only resveratrol-induced post-translational modification of p53 as shown above, but also DNA binding by COX-2/p53.

**Figure 7:**
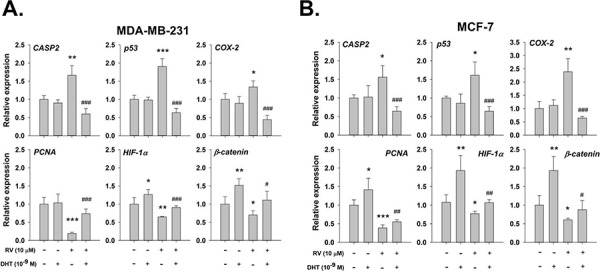
DHT inhibits resveratrol-induced pro-apoptotic gene expression **A.** MDA-MB-231 cells **B.** MCF-7 cells were treated with 10 μM resveratrol in the presence or absence of 10^−9^ M DHT for 24 h. Cells were harvested and total RNA was extracted for QPCR as described in the Materials and Methods section. DHT inhibited the RV-induced gene expressions of *CASP2*, *p53* and *COX-2*. The resveratrol-suppressed gene expressions of *PCNA*, *HIF-1α* and *β-catenin* were enhanced by DHT. *N* = 4. *p < 0.05; **p < 0.005; ***p < 0.001, compared to control. # *p* < 0.05; ## *p* < 0.01; ### *p* < 0.001, compared between resveratrol-treated samples in the presence and absence of DHT.

To determine the functional significance of the ChIP results, QPCR studies were carried out. In our studies of transcription by QPCR in MDA-MB-231 cells, resveratrol induced expression of *CASP2, p53*, and *COX-2* (Fig. 7A). On the other hand, DHT, 10^−9^ M, alone stimulated the expression of *HIF-1α* and *β-catenin*, but not *PCNA*. When cells were pretreated with DHT, the resveratrol-inhibited genes expression were reversed (Fig. 7A). Similar results of inhibitory effect were observed in resveratrol-treated MCF-7 cells (Fig. 7B).

## DISCUSSION

We demonstrate that DHT interfered with resveratrol-induced anti-proliferation in both ER-α-positive MCF-7 and ER-α-negative MDA-MB-231 human breast cancer cells. The role of ER-α in the proliferative effect of DHT in MCF-7 cells is demonstrated by the actions of the specific inhibitor of ER, ICI 182,780 to block DHT-induced proliferation [11]. In MDA-MB-231 cells, a novel mechanism was implicated, in that anti-integrin αvβ3 or an Arg-Gly-Asp (RGD) peptide targeted at a binding domain of the integrin each eliminated the DHT effect on cell proliferation [11]. Treatment of AR-positive prostate cancer LNCaP cells with resveratrol induces a decrease in receptor levels that is more pronounced at the highest concentration used of 150 μM [39]. In an androgen-insensitive prostate cancer PC-3 cell line not expressing AR but having ER-α [40], resveratrol induces reduction in ER-α with a maximum effect at 100–150 μM [39]. However, the concentration of resveratrol used in our studies did not exceed 10 μM, thus there may be other inhibitory mechanisms involved in the interference of DHT on resveratrol-induced anti-proliferation in breast cancer cells.

In DHT concentration–response studies, there was hormone concentration-dependent enhancement of resveratrol-stimulated ERK1/2 activation with 10^−10^–10^−8^ M DHT (Fig. 3A) but reduction in resveratrol-induced Ser-15 phosphorylation of p53 after treatment with both agents for 4 h. This latter effect was also DHT concentration-dependent. The effect of 10^−10^–10^−8^ M DHT on resveratrol-induced anti-proliferation in MDA-MB-231 cells and MCF-7, indicated by nucleosome ELISA assay and MTT assay (Fig. 3B and 3C). Dose-responsive inhibition of resveratrol-induced apoptosis by DHT in these resveratrol-treated cells directly correlates with the reduction in the Ser-15 phosphorylation of p53 (Fig. 3A).

Although resveratrol has been shown by others to have estrogen-like activity in a transcriptional assay in cells expressing predominantly ERα [32, 41]. ICI 182,780, in a relatively low concentration of 3 nM, does not reduce resveratrol-induced activation of ERK1/2 and serine phosphorylation of p53 [38]. Since DHT action is inhibited by ICI 182,780 in MCF-7 cells, while resveratrol activity is not affected by ICI 182,780 (Fig. 4), the inhibitory effect of DHT on resveratrol-induced serine phosphorylation of p53 is interpreted to be ER mediated. On the other hand, both resveratrol- and DHT-induced activities were inhibited by anti-integrin αvβ3 antibody in ER-α negative breast cancer cells (Fig. 5).

Since the effect of resveratrol that we have demonstrated in breast cancer cells focuses on the modification and action of the tumor suppressor p53, it is conceivable that cancer cells harboring mutant p53 might not respond in the same manner. However, studies by Hsieh *et al*, have documented resveratrol-induced reduction in growth of MDA-MB-435 cells, which contain a mutant p53 [42]. In our studies of the actions of resveratrol on DU145 prostate cancer cells, which also contain mutant p53, resveratrol did induce apoptosis [43]. Future studies are to be directed towards the identification of critical sites on p53 for resveratrol action.

Treatment of human breast cancer cells with 10 μM resveratrol in the presence of 10^−9^ M DHT produced additive ERK1/2 activation by DHT and resveratrol. However, DHT inhibited resveratrol-induced phosphorylation of Ser-15 in p53, despite the enhancement by DHT of ERK1/2 activity. The downstream consequence of ERK1/2 activation by DHT alone did not induce apoptosis as other steroid hormones do [29, 38], compared with the actions of resveratrol. Thus, although both resveratrol and DHT induce ERK1/2 activation, divergent effecs are observedin the downstream ERK1/2 on nuclear transcription of COX-2 and p53 by resveratrol and blocking apopotosis by DHT. This is consistent with the existence of discrete intracellular pools of ERK1/2 regulated by resveratrol and DHT separately. The anti-apoptotic effect of DHT in the presence of resveratrol involves rapid stimulation of signaling cascades, including the ERK1/2 pathway. In our studies, the ER inhibitor ICI 182,780 suppressed both DHT-induced ERK1/2 activation and the inhibitory effect of DHT on resveratrol-induced Ser-15 phosphorylation of p53 (Fig. 4). Similar observation is seen in the effect of thyroid hormone-inhibited resveratrol-induced anti-proliferation in glioma cells by interfering with the interaction of nuclear COX-2 and ERK1/2 [29]. This finding is consistent with DHT inhibition of post-translational modifications of p53 that are essential for p53-dependent gene transcription.

Androgen-induced ERK1/2 activation, in contrast to ERK1/2 activation which is also observed in resveratrol, will not cause apoptosis, and we do not expect that androgen will cause p53 phosphorylation. We have shown that another steroid hormone, E_2_ inhibits resveratrol-induced p53-dependent apoptosis by interfering with p53 binding on promoter and p53-dependent p21 expression [38]; therefore, it is not surprising to observe that DHT performs the same way to block COX-2 binding to promoters in ChIP assay and p53-dependent gene expression (Fig. 6C and Fig. 7). DHT alone had no effect on the binding of p53 to a relevant oligonucleotide. However, DHT did inhibit resveratrol-induced binding of p53 to the oligonucleotide (Fig. 6C). In our previous study, resveratrol has been shown to highly upregulate genes supporting anti-proliferation such as *CASP2* and genes that support apoptosis, e.g., *BAD*, *p53*, *TP53I3*, *p21*, *c-fos* and *COX-2* [31]. We also showed that resveratrol induced the expression of *COX-2*, *CASP2* and *p53*, but inhibited the expression of *PCNA*, *HIF-1α* and *β-catenin* (Fig. 7) which play roles in cancer proliferation and metastasis.

The present studies indicate that ambient DHT may antagonize actions of resveratrol in selected treatment paradigms. Our results suggest that the complexity of actions of resveratrol might occurr in both *in vivo* and *in vitro* settings. When resveratrol is tested in intact animals, it would be important to control for levels of androgen, and useful to examine actions of the nature stilbene in the presence of a DHT antagonist. As indicated above, we know that both DHT and resveratrol have receptor sites on integrin αvβ3 in ER-negative breast cancer cells and that both hormone and stilbene activate ERK1/2. However, it is difficult to demonstrate the existence of discrete hormone- and stilbene-binding sites on integrin αvβ3 in ER-α negative breast cancer cells, although the antagonism is raised by DHT in the action of resveratrol on signaling events that promotes apoptosis is clear.

**Figure 8:**
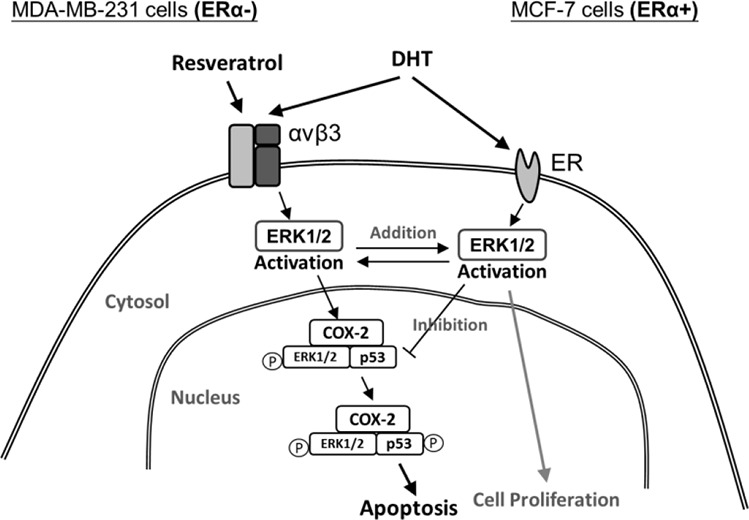
Signal transduction pathways involved in resveratrol-induced apoptosis and in inhibitory effects of DHT on actions of resveratrol in ER-α-positive and negative breast cancer cells DHT binds to membrane ER-α in ER-α-postive breast cancer cells and integrin αvβ3 in ER-α-negative breast cancer cells. DHT activates ERK1/2 and induces cell proliferation. Resveratrol binds to integrin αvβ3 activates ERK1/2, which is essential for resveratrol-induced expression of COX-2. The newly synthesized COX-2 is associated with phosphoERK1/2 translocates to the nucleus where the complex binds to p53, in the course of which COX-2 is sumoylated and p53 is phosphorylated and acetylated. The COX-2 and p53 complex binds to promoters of p53-responsive genes and initiates transcription. The activation of ERK1/2 by resveratrol and DHT are separate processes that do not interferewith each other. However, the activation of ERK1/2 by nonpeptide hormones is essential to the inhibition of apoptosis induced by resveratrol. DHT inhibits resveratrol-induced cellular activities as indicated. P, phosphorylation.

In summary, DHT via distinct binding receptors in ER-positive and negative breast cancer cells, ERα in ER-positive and integrin αvβ3 in ER-negative breast cancer cells activated ERK1/2 and further stimulated cell proliferation (Fig. 8). On the other hand, resveratrol bound to integrin αvβ3 to activate ERK1/2, nuclear COX-2 accumulation and phosphorylated p53 and COX-2 complexing which further induced expression of pro-apoptotic genes and anti-proliferation (Fig. 8). All the actions downstream the ERK1/2 activation were targeted by DHT for interference. Results presented here indicated that it is possible to use resveratrol therapeutically in breast cancer treatment. However, in addition to estrogen, DHT may be another interfering factor for resveratrol therapy and resveratrol may be most effective in a DHT-depleted environment. In addition, it may be clinically practicable to treat ER positive breast cancer patients with resveratrol in the present of ICI or tamoxifen to block the interference with DHT and estrogen. The usage of resveratrol therapy in ER-negative breast cancer patients may raise more concern than expected in the future.

## MATERIALS AND MEHTODS

### Cell lines

Human breast cancer MCF-7 (ER-α-positive, ATCC^®^ HTB-22^™^) and MDA-MB-231 (ER-negative, ATCC^®^ HTB-26^™^) cells were purchased from American Type Culture Collection (ATCC, Manassas, VA, USA) by Bioresource Collection and Research Center (BCRC, Hsinchu, Taiwan). These two cell lines had been tested and authenticated (including isoenzyme analysis, Mycoplasma test, cytogenetics test, tumorigenic test and receptor expression test) by BCRC. We purchased them from BCRC and passaged in our laboratory for fewer than 6 months after thawing and maintained for further study in Dulbecco's Modified Eagle Medium (DMEM, Life Technologies Corporation, Carlsbad, CA, USA) supplemented with 10% FBS and incubation conditions are 5% CO_2_ at 37°C. Before the study, cells were placed in 0.25% hormone-depleted serum–supplemented medium for 2 days.

### Western blotting

To examine the effects of DHT in resveratrol-induced anti-proliferative signaling pathways, we performed western blot analysis to quantify the protein expression levels of pERK1/2, pSer15-p53, and pPI3K(p85) in the total cell lysates of MCF-7 cells and MDA-MB-231 cells which were treated with 10 μM resveratrol and/or 10^−9^ M DHT. Protein samples were resolved on a 10% Sodium Dodecyl Sulfate polyacrylamide gel (SDS-PAGE). A 20-μg quantity of protein was loaded in each well with 5x sample buffer, and the protein samples were resolved by electrophoresis at 100 V for 2 hrs. The resolved proteins were transferred from the polyacrylamide gel to Millipore Immobilon-PSQ Transfer PVDF membranes (Millipore, Billerica, MA, USA) with the Mini Trans-Blot^®^ Cell (Bio-Rad Laboratories, Inc., Hercules, CA, USA). The membranes were blocked with a solution of 2% bovine serum albumin in Tris-buffered saline. The membranes were incubated with primary antibodies to phospho-p44/42 MAPK (pERK1/2), ERK2, pSer15-p53, p53, pPI3K(p85), PI3K (Cell Signaling Technology, Inc., Beverly, MA, USA), β-actin (GeneTex International Corporation, Hsinchu City, Taiwan), Lamin B1 (Abcam, Cambridge, MA, USA), at 4°C overnight and washed, and the proteins were detected with HRP-conjugated secondary antibodies and ImmobilonTM Western HRP Substrate Luminol Reagent (Millipore). Images of the Western blots were visualized and recorded by BioSpectrum^®^ Imaging System (UVP, LLC, Upland, CA, USA).

### Nuclear protein extraction and immunoprecipitation

To examine whether DHT affects the resveratrol-induced nuclear accumulated COX-2 and forms complex with phosphorylated p53. Nuclear and cytoplasm proteins were extracted and isolated from MDA-MB-231 cells by means of the Thermo Scientific NE-PER^®^ Nuclear and Cytoplasmic Extraction Kit protocol (Thermo Scientific, Rockford, IL, USA). The cells underwent lysis in cytoplasm extraction reagent and were centrifuged at 16,000 × g for extraction of the nuclear material. Proteins from the nuclear material were extracted by the addition of nuclear extraction reagent to the nuclei and centrifugation at 16,000 × g. Nuclear extracts were stored at −80°C until use. The protein concentration in the nuclear extracts was measured with the BCA^TM^ Protein Assay kit (Thermo Scientific). Samples containing 200 μg nuclear proteins were immunoprecipitated with the rabbit anti-COX-2 antibody (Abcam) or p53 (Cell Signaling Technology) followed by Western blotting with antibodies of anti-pSer15-p53, p53, or COX-2. The anti-Lamin B1 antibody (Abcam) was used as loading control.

### Confocal microscopy

MDA-MB-231 breast cancer cells were exponentially growing on sterilized cover glass (Paul Marienfeld GmbH & Co. KG, Lauda-Königshofen, Germany) and treated with anti-integrin αvβ3 antibody (Santa Cruz Biotechnology, Inc., Dallas, TX, USA) for 24 hr prior to the treatment of either resveratrol, DHT or the combination for another 24 hr. The samples were immediately fixed with 4% paraformaldehyde in phosphate buffered saline (PBS) for 10 minutes. Cells were permeabilized in 0.1% Triton X-100 in PBS for 20 minutes. The cells on the slides were incubated with anti-phosphoPI3K antibody or anti-pSer-15-p53 antibody (Cell Signaling Technology, Inc., Danvers, MA, USA) overnight in 4°C. Then the cells were incubated with tetramethylrhodamine isothiocyanante (TRITC)-conjugated secondary antibody (Abcam, Cambridge, United Kingdom) and a Hoechst stain for nucleus. The red fluorescent signals from phosphoPI3K and phospho-Ser15-p53 were recorded and analyzed with TCS SP5 Confocal Spectral Microscope Imaging System (Leica Microsystems, Wetzlar, Germany). The figures shown are representative of four fields for each experimental condition.

### Chromatin immunoprecipitation

A total of 6 × 10^6^ MDA-MB-231 cells were exposed to 1% formaldehyde for 10 min at room temperature to effect crosslinking and then added glycine to a final concentration of 125 mM for 5 min to stop crosslinking. Monolayers were washed twice with cold PBS and cell extracts prepared by scraping cells in 1 mL of buffer (150 mM NaCl, 1% NP-40, 0.5% DOC, 0.1% SDS, 50 mM Tris [pH 8], 5 mM EDTA) containing leupeptin (10 μg/mL), pepstatin A (10 μg/mL), NaF (50 mM), 0.2 mM sodium orthovanadate and trichostatin A (5 μM; Calbiochem, San Diego, CA, USA). Cell lysates were sonicated to yield chromatin fragments of approximately 200 - 500 bps, as determined by 2% agarose gel electrophoresis. Sonicated lysates were then diluted to 2 mL with ChIP dilution buffer (0.01% SDS; 1.1% Triton X-100; 1.2 mM EDTA; 16.7 mM Tris-HCl, pH 8.0; and 167 mM NaCl), followed by preclearing with 80 μL protein A–agarose beads/protein G Plus–agarose beads for 60 minutes at 4°C with rotation. 50 μL precleared lysates of each experiment were removed and then extracted DNA as INPUT DNA. The precleared lysates were next immunoprecipitated using antibodies for COX-2 (4 μg; Abcam) at 4°C overnight with rotation. The no-antibody control was also included with each experiment. Immune complexes were collected with 60 μL protein A–agarose/protein G Plus–agarose and washed once with 1 mL each of the following buffers: low salt wash buffer (0.1% SDS; 1% Triton X-100; 2 mM EDTA; 20 mM Tris-HCl, pH 8.0; and 150 mM NaCl), high salt wash buffer (0.1% SDS; 1% Triton X-100; 2 mM EDTA; 20 mM Tris-HCl, pH 8.0; and 1500 mM NaCl); and twice with 10 mM Tris-HCl (pH 8.0) and 1 mM EDTA. Immune complexes were next eluted using freshly prepared elution buffer (1% SDS and 0.1 M NaHCO_3_). Cross-links were reversed by heating at 65°C in the presence of NaCl followed by proteinase K treatment. The DNA was recovered by PCR-purification spin column (Qiagen, Hilden, Germany) and resuspended in 50 μL distilled water. To examine whether p53 responsive DNA elements were binded with COX-2/p53 complexes, ChIP DNA was next used as a template for quantitative real-time polymerase chain reaction (QPCR) by using the appropriate primers: *PIG3*, 5′-CAGGACTGTCAGGAGGAGGCGAGTGATAAG G-3′ (forward) and 5′-GTGCGATTCTAGCTCTCACTTCAAGGAGAGG-3′ (reverse) and *Bax*, 5′-TAATCCCAGCGCTTTGGAAG-3′ (forward) and 5′-TGCAGAGACCTGGATCTAGC-3′ (reverse). Calculations of promoter DNA expression were performed as described previously [44, 45]. We also performed PCR and the PCR product was resolved using 2.5% agarose gels in 0.5X Tris-borate-EDTA buffer. Gels were stained with SYBR Safe (Thermo Fisher Scientific Inc., Waltham, MA USA), then were visualized and recorded by BioSpectrum^®^ Imaging System (UVP, LLC, Upland, CA, USA).

### Quantitative real-time PCR

To examine mRNA expression, we treated MCF-7 cells and MDA-MB-231 cells with vehicle, 10^−9^ M DHT, 10 μM resveratrol or combined treatments for 24 h. Total RNA was extracted and genomic DNA was also eliminated with illustra RNAspin Mini RNA Isolation Kit (GE Healthcare Life Sciences, Buckinghamshire, United Kingdom). 1 μg of DNase I-treated total RNA was reverse-transcribed with RevertAid H Minus First Strand cDNA Synthesis Kit (Life Technologies Corporation, Carlsbad, California, USA) into cDNA, and used as the template for real-time PCR reactions and analysis. The real time PCR reactions were performed using QuantiNova^TM^ SYBR^®^ Green PCR Kit (QIAGEN) on CFX Connect™ Real-Time PCR Detection System (Bio-Rad Laboratories, Inc., Hercules, CA, USA). This involved an initial denaturation at 95°C for 5 min, followed by 40 cycles of denaturing at 95°C for 5 sec and combined annealing/extension at 60°C for 10 sec, as described in the manufacturer's instructions. The primer sequences were shown as following: human caspase 2, apoptosis-related cysteine peptidase (*CASP2*), forward 5′-GCATGTACTCCCACCGTTGA-3′ and reverse 5′-GACAGGCGGAGCTTCTTGTA-3′ (Accession No.: NM_032982.3); Homo sapiens tumor protein p53 (*p53*), forward 5′-AAGTCTAGAGCCACCGTCCA-3′ and reverse 5′-CAGTCTGGCTGCCAATCCA-3′ (Accession No.: NM_000546.5); Homo sapiens cyclooxygenase 2 (*COX-2*), forward 5′-GCCAAGCACTTTTGGTGGAG-3′ and reverse 5′-GGGACAGCCCTTCACGTTAT-3′ (Accession No.: AY462100.1); Homo sapiens proliferating cell nuclear antigen (*PCNA*), forward 5′-TCTGAGGGCTTCGACACCTA-3′ and reverse 5′-TCATTGCCGGCGCATTTTAG-3′ (Accession No.: BC062439.1); Homo sapiens hypoxia inducible factor 1, alpha subunit (*HIF-1α*), forward 5′-TGAACGTCGAAAAGAAAAGTCTCG-3′ and reverse 5′-GGAAGTGGCAACTGATGAGC-3′ (Accession No.: NM_001243084.1); Homo sapiens catenin (cadherin-associated protein), beta 1, (*β-catenin*), forward 5′-CTGGTCCTTTTTGGTCGAGGA-3′ and reverse 5′-GCAAGGCTAGGGTTTGATAAAT-3′ (Accession No.: NM_001904); human glyceraldehyde-3-phosphate dehydrogenase (*GAPDH*), forward 5′-TGCCAAATATGATGACATCAAGAA-3′ and reverse 5′-GGAGTGGGTGTCGCTGT TG-3′ (Accession No. NM_002046). Calculations of relative gene expression (normalized to GAPDH reference gene) were performed according to the ΔΔCT method. Fidelity of the PCR reaction was determined by melting temperature analysis.

### Apoptosis/nucleosomes

Cells were treated with different reagents for 48 h. The cells were harvested, spun down and pellets were washed twice with phosphate-buffered saline. Pelleted cells were then lysed and the supernatants were collected and stored for at least 18 h at −20°C. From each appropriately diluted sample, 100 μl were added to a 96-well plate coated with a DNA binding protein and incubated at room temperature for 3 h. After three washes with buffer, detector antibody was added for 1 h. Streptavidin conjugate was then added and incubated for 0.5 h before adding substrate. Plates were read at 450 nm. The nucleosome ELISA kits for these studies were purchased from Calbiochem.

### Cell viability assay

MCF-7 cells (5 × 10^3^ cells per well) were seeded in 96-well plates. After starvation with serum-freem medium for 48 h, cells were then treated with vechicle, 10 μM RV, 10^−10^M, 10^−9^M, 10^−8^M DHT or combined treatment for 96 hr. Cell proliferation was determined by incubating the cells with 200 μl of fresh medium containing 1 mg/ml 3-(4,5-dimethylthiazol-2-yl)-2, 5-diphenyltetrazolium bromide (MTT) (Sigma-Aldrich) for 4 h at 37°C. After removal of the MTT solution, the resulting formazan crystals were dissolved completely in dimethyl sulfoxide and the plates were read using a microplate reader (Anthos 2010; Biochrom, Cambridge, United Kingdom) by measuring the absorbance at 540 nm. Quintuplicate wells were assayed for each experiment and three independent experiments were performed. Data are expressed as the mean ratio (%) of OD540 ± SD which compared with vehicle.

### Data analysis and statistics

Immunoblot and nucleotide densities were analyzed by IBM^®^ SPSS^®^ Statistics software version 19.0 (SPSS Inc., Chicago, IL, USA). Two tails Student's *t-test* was conducted and considered significant at *p*-values < 0.05 (*, or #), 0.005 (** or ##) and 0.001 (***, or ###).
